# Assisted Suicide

**Published:** 2010-03-31

**Authors:** Constantin Dan Manu

**Affiliations:** Forensic-medicine specialist, County Hospital of Piteşti, Romania

## The suicide definition

The **Oxford** Dictionary defines the English noun “suicide” as:

1. person who intentionally kills himself
2. intentional self-slaughter; commit suicide –kill oneself intentionally. The term has a Latin origin: sui – of oneself-reflexive pronoun singular- and the verbal form –cidium- from the verb to kill.

The **Random House Webster** Dictionary defines the term as:

1. person who intentionally kills himself
2. the intentional taking of someone’s own life; to commit suicide – to kill oneself

The Modern Romanian Language Dictionary defines the noun “suicide” –the suicidal action; to kill himself

The Neologisms Dictionary defines the noun “suicide” – the action of killing oneself and its result –the suppression of one’s own life; suicide.

Out of didactical and specialty reasons in legal medicine and psychiatry, supplementary explanations are given to the Romanian term “sinucidere”, which is more and more used as “suicid”, considered a form borrowed from the French by the Neologisms Dictionary.

The definition of the term is the following:

The act of taking the personal life of a subject, who is not ailed by an affliction that condemns him in an inexorable way to death, independently of his will.

The specifications are made especially in order to separate the situations of the suicide of the person who is already condemned by his fate.

The suicide is generally considered the expression of a pathological mental state, commonly a depression. A British study conducted in 1974, which included extended interviews and the analysis of the observation sheets, concluded that those who survived a suicidal attempt were mentally ill at the time of the act. Another study conducted in St.Lois in 1984, found mental disorders in 94% of those who attempted a suicide. The most of them acted as if under an unconscious call for help and not like after a cool conclusion that death is preferable to life.

The psychologist Joseph Richman, a clinic suicide specialist and a psychotherapist stated in “The suicide diary and the behavior that threatens life” that: “I was impressed by those persons who have suicidal tendencies because they are different to anybody else, including those who select the rational suicide.”

Today there is a questionnaire form called DR. SCISORS (Death Readiness: Short Clinical Indicator Scale of Rational Suicide), sounding like the English word “scissors”. This is used in clinics in order to make a distinction between the reasonable and the irrational suicide.

Anyhow, the sane persons who have suicidal attempts must be helped to solve their problems and not be supported in their self-destructive tentative.

In the case of the persons who suffer from chronical irreversible afflictions with a lethal forecast, sometimes the suicide may appear as self- deliverance. The reactional suicidal attempts or the psychotic depressive or paranoic suicides are excepted.

## The difference between suicide and voluntary euthanasia

**The difference between common suicide and appealing to voluntary euthanasia consists in the presence of an incurable and/or lethal affliction**.

Even the Dutch doctor Pieter Admiraal, the leader of the successful movement for the legalization of the euthanasia in Holland, stated in public that **pain is never a proper justification for suicide**, due to the medical technologies that can result in a pain management in almost all the cases.

The lust for life, on the other side, is compatible with the request of getting an easy death. For example, a young AIDS patient would like to live, but cannot bear the idea of physical and mental disability given by this illness quite a long time before dying. Therefore, he may decide to hasten the moment of death, avoiding this time lapse.

### A short history of the attitude toward suicide

Among the precepts of the religious morale, the wow to keep one’s life enjoyed an absolute authority. That is why suicide was considered an act of indignity, a sin of man in front of God. In some middle age German states the suicide was attached to a horse, head down and dragged out of town.

The Christian tradition regarding all forms of suicide was well documented by:

Saint Thomas D’Aquino (aprox. 1225-1274 A.D.). He condemned the suicide using three principles:

- Life is but a gift from God and only God can take it back
- Suicide is against the natural will to live
- Suicide afflicts other persons too

**Dante Aligheri (1265-1321)** in “The Divine Comedy” states that: The suicide must bear all the torments of Hell.”

**Michel de Montaigne** (1533-1592), French writer, former mayor of Bordeaux, who published in 1580 a set of “Essays”, in which a stoic wisdom and sometimes a skeptic one as well is obvious, refuses such ideas. He wrote five essays treating the subject of suicide, where he says:

- Suicide must be considered a matter of personal option
- Suicide is a rational option in some circumstances

As the values lost their religious content the tradition regarding the ban on suicide did not disappear, it only changed the motivation on its banning. As the duty towards oneself and the society compels the man to pay respect to his own life, suicide shifted from the guilt in front of God to the social crime and a moral guilt in front of oneself.

Louis the XIVth (1638-1715) considered suicide as an act of “lese majeste” (i.e. an insult to the Crown), therefore he refused the right of burial for the suicides.

**Matei Basarab** wrote in the “Law” of 1652 that: He that takes his life at his own decision, he is not to be remembered nor given the religious services, because he has sold his soul to Satan.

**Jean-Jaques Rousseau** (1712-1778) the French speaking philosopher of Geneva, who in his life was a wanderer, abandoned his own five children in a Boarding House, contributed with some texts to the great (French)”Encyclopaedia”, pleaded for democracy in his book the “Social Agreement”, described the suicide as a “ robber’s death and a shameful one…a theft against the human species”

**Immanuel Kant**, ( 1724-1804), the German philosopher who dedicated his life to knowledge and to teach the others at the Koenigsberg University, brought convincing arguments against the rare cases of “rational suicides” in his essay “ Lectures on Ethics”. His ideas are the following:

- Suicide is incompatible with respect to somebody’s humanity, due to the scholarly consideration as “theological intent” in order to avoid pain or suffering.
- Suicide is contradictory in itself, due to the fact that the power of free will (the right to choose) is used for self-destruction.
- Suicide degrades the human value by bringing it to the animal level or even lower.
- Suicide is against the highest duty of the human being towards itself – the self-respect as a person.

## The moral and psychological arguments on suicide in the past and in the present days

**Auguste Comte** (1798-1857), the French philosopher who founded the positivist school, considering that the human spirit (be it of an individual or of a civilization) necessarily passes from the theological stage to the metaphysical one in order to rise to the positive stage, he was considering the suicide as lacking morality, because: “At the positive age there is only an absolute maximum, that is, there is nothing absolute.”

**Emile Durkheim** (1858-1917), the French sociologist who was influenced by positivism, who defined the object and the methods of sociology, wrote a magnificent paper entitled exactly “Le suicide”(1897), where regarded from the social viewpoint, the act of suicide is viewed as imoral:

“The state of deep disturbance that afflicts the civilized society is proved by the exceptional great number of suicides; this confirms its seriousness as well. One can say that it also offers its measure. We can light up this flow of collective sadness only by softening the collective illness that is its own result and sign.”

Therefore, the self-preservation is looked upon as an absolute duty towards self and society.

The Roman Church totally banns the funeral service and the material and moral support to those who have committed a suicidal act or to their relatives. Reminiscences of this kind continued up to our time, suicide being considered a criminal offence, as an ecclesiastic sin, although these attempters needed in fact some help without being punished. The Christian Moral that refused the suicides and the religious assistance were not able to stop these self-slaughter acts after all.

The XXth Century moralists separated the suicide from the idea of guilt; this has been considered only a psychological drama and an intimate tragedy. As the self-slaughter act does not mean an abdication from the moral duty anymore and does not attract the collective blame, the suicide is perceived only as a personal tragedy raising compassion and questioning over the motivations rather than getting disapproval.

**Khalil Gibran** (1883-1931), the mystical Lebanese poet and painter, who founded an Arabic literary society in New York, wrote in his monumental poem “The Prophet”:

“You would like to know the secret of death. However, how to find it if you neglect the heart of life? For life and death are just one thing, as the river and the sea.”

## The right not to suffer

The contemporary situation

Now, as we dispose of so many pharmaceutical facilities and modern technologies unconceivable in the recent past (analgesics, antibiotics, instruments, and so on and so forth) the physical suffering is beginning to be intolerable and unfair.

Therefore, by the extension of the subjective rights the collectivity sometimes justifies the right of the individual to hasten its own death implying the right not to suffer. The duty to keep our own life was replaced by the right to be a master over one’s own life. Everyone has the right to autonomy, and suicide has no longer been considered a crime in many states for a long time.

## The right to autonomy

Instead of the right to autonomy, most of the states promote protective measures and legitimate forbidding ones, by which the society strives to hamper the self-destruction of its members.

Nevertheless, an ever-growing number of citizens from different parts of the world ask for the legitimating of the medical assisted suicide in the name of the human dignity. They consider that, as every human being has a legitimate right regarding its own death and the manner of this death, the medical assisted suicide is a last act of freedom of the man who refuses the show of his own decay and degrading.

## The conflict between the life options and a dignified death

Some pro-choice groups, which support the right to choose, tried to show horrible terminal ill persons, suffering from unbearable pains that are untreatable, in spite of the fact that such cases do not represent the general case.

Scottish and American societies that are militant for the right to die, made financial efforts to publish “Self-deliverance Guides” that sometimes proved an unexpected sale success.

In 1980, The Scottish Exit Society, which is now called The Voluntary Euthanasia Society of Scotland – VESS- published “How to Die with Dignity” –the first Guide for suicide in the world. The book was not sent to the bookstores but only via personal order by post or television.

In 1981, The Hemlock Society issued “Let Me Die Before I Wake” the first suicide guide sold freely in bookshops.

In 1991, The Hemlock Society issued “Final Exit: The Practicalities of Self-Deliverance and Assisted Suicide for Dying”, considered a best seller – in less than a half a year 540000 copies were sold- Final Exit was translated in 12 foreign languages, and the total sales went over a billion copies.

In 1993, The Voluntary Euthanasia Society of Scotland –VESS- reprinted the first guide “How to Die with Dignity”, adding a supplement named “Departing Drugs”. The supplement was written in compliance with the International Drug Consensus Working Party scientific researches, being reprinted in several worldwide spoken languages.

In 1995, The Voluntary Euthanasia Society of Scotland (VESS) issued a last self-deliverance guide “Beyond Final Exit” updated to the last researches in the field. Based on hundreds of referrings and stories, the guide contains the result of the work of some researchers all over the world, offering data checked in practice and facts on suicidal methods. The guide cannot be purchased in bookstores, but can be ordered by post or Internet in Europe, and with some difficulty in the USA and Canada.

In 1991, the Pittsburg Legal Medicine Bulletin, entitled “Scalpel and Pen” suggested as meditation topic the following argument:

“I am not afraid of dying, I am afraid of illness, and of what it can do to me. I cannot defeat it; I am more and more ill. There is no consolation. Nothing, only loathing and pain...Who benefits of my dying slowly? I stay between life and death and I wish I did not go on like this at all. I see no reason not to leave it. (“The Last Wish”).

### The difference between assisted suicide and other forms of euthanasia

Only France is the country where it is illegal to offer information on suicide. “Assist” regarded as to be witnessed without taking any action, may be considered as illegal in some countries. Controversy appears when “to assist” means to directly offer means for suicide.

Along with practice, the interpretation of the term swelled, even included the administration of the lethal substance by a doctor to the suffering patient – a kind of euthanasia.

The assisted suicide can be defined as a kind of mixed voluntary euthanasia (active and passive), although some speak only of voluntary passive euthanasia –in short VPE.

Assisted suicide is a legal practice in Switzerland, Holland, Oregon state in USA and was legal for a while in the Northern Territory of Australia.

## Defining the term suicide assisted by a doctor

**The suicide assisted** by a doctor is**:**

Generally, in the practice of the assisted suicide the doctor prescribes the medicine that implies death and assists the patient in his intention to end life. That is why one uses the term “physician assisted suicide”, which is already known and quoted in short PAS on most of the internet web sites.

Unfortunately, both the supporters of the assisted suicide and its most violent opponents recurred to different intimidation tactics, but unfortunately, these can only be used for short periods of time in order to modify the public attitude.

This phenomenon that entered the daily practice of different social activists is also a subject of academic debates in the USA. Its practice, legally implemented only in the state of Oregon, stirs up the wish to be adopted by other citizens of the American states, where, for the time being, there is a legal interdiction. In 37 states, the interdiction is stated in specific law texts, and in other eight states it is prohibited according to the common right legislation or statements on murder.

In 26 American states, law projects regarding assisted suicide have been entered in 1997 and 1998 and have been rejected. The inhabitants of the states of Michigan and Washington have rejected the pooling initiatives on some laws that intended to legalize the assisted suicide and, in Michigan, Virginia, North Carolina, Iowa and Rhode Island new obstacles were set. As the Supreme Court of USA decided that there is no constitutional right to assisted suicide, the states are free to condemn it, everyone in turn. In the same time, they are also allowed to admit it, as the state of Oregon has already done it.

The improper medical care level for those about to die and the enhancing of the quality of life for those with severe disabling diseases who are not dying, maintain the problem of legalization in the attention of doctors, lawyers, moralists, press people and the general public. The jury of Michigan that found Dr. Jack Kevorkian guilty rejected his plea, while he was defending himself and desired to wipe the demarcation line between assisted suicide and euthanasia. In spite of the legal decision, he had a considerable public support, and the efforts towards legalization of assisted suicide continued under a very good organization. The national policy and the American legal agenda have been preoccupied with the Pain Relief promotion Act of 1999, and the doctors lived moments of maximum efforts being in the centre of the debates.

In 1997, The Bioethics Center of the Pennsylvania University brought together a Multi-disciplinary Collective of National Experts on Assisted Suicide. Its members were hospital and mental house doctors for palliative medicine, nurses, psychologists, lawyers, members of patient associations, clergymen and bioethicians. The selection was deliberately heterogeneous, containing different opposing points of view, both pro and cons ones. The discussions started from the hypothesis of the legalization of the assisted suicide, as it happened some months later in the state of Oregon, in order to find out a way for those who wanted to benefit from such a legal provision. The members of the team desired to establish some limits of guarantee that the assisted suicide will always be voluntary, well regulated and considered a last option.

The project “Finding Common Ground” that established an Assisted Suicide Consensus Panel, which had to answer the following questions, was conceived in the University of Pennsylvania:

- What is assisted suicide?
- Is the doctor assisted suicide different from the refuse of treatment?
- What would be the alternatives to these?
- How useful are the current available guides for assisted suicide?
- Does the assisted suicide mean necessarily a doctor assisted suicide?
- Can the assisted suicide be regulated in a significant and efficient way?
- When are we to come to palliative treatments as a last resource?
- How must the doctors answer to the patients request to be helped to die?

The members of the team did not agree on all terms due to the different point of views, but many consensus elements were discovered.

According to the Oregon laws, **the resident citizens there**, who are suffering from a terminal illness and are healthy from the mental point of view, can receive a receipt from a doctor, with which they may obtain a lethal dose of medicine, after they have applied in writing and orally for a certain period and followed some procedures.

**The doctors** who assist these patients must fulfill in return a long list of conditions. A year after the application of the law the practical consequences are made public – the number and the ages of those who benefit from it, the illnesses and the motivations for this ending. It seems that the fear of pain was less of a motive compared to the preoccupation of not losing the control and the autonomy of the self.

**The supporters of the law** show that there was no rush to benefit from the advantages offered by it, and no abusive usage was recorded.

**Those who opposed it** think that not all the cases were reported. They also stress the fact that this practice was requested not only for the reasons mentioned in the text of the law, i.e. the pain and the suffering but also for other ones.

In March 2000, the conclusions were given to the press. As a result of the debates and discussions, **five documents** have been published.

In the first, one of the conclusions of the team was: “The assistance given by the doctor was not enough to grant that the assisted suicide is limited only to the appropriate cases and that it takes place accordingly.”

**Although the doctors are necessary** in order to diagnose the terminal illness, to prescribe the medicine and to fill in the death certificate, other tasks, such as the orientation of the pressure, the spiritual matters and the noticing and controlling of the symptoms**, imply only a limited competence**.

**Barbara Coombs Lee**, Executive Officer of the Federation “Compassion for those about to Die” from Portland, Oregon state, sustains that it is impossible to do such a thing because the help to die is illegal. She stated:

“According to my opinion, this is a main argument in the favor of legalization. Instead of hiding and being mysterious, we could be open and kind, bringing forth the applications before the most illuminated minds and acting as precautious as in the moment we take the life termination decisions, such as the disconnection from the oxygen units, administration of the terminal sedatives or the interruption of the artificial water and food supply.”

In the second document, the authors draw the conclusion that:

“The legalization implies extra responsibilities for doctors, whatever their opinions regarding the assisted suicide.”

Doctor James Tulsky, Associate Medical Professor at the Medical Center of Duke University in Durham, sustains that:

“The obligation to answer to an absolutely desperate person, who asks such a thing, must not be transformed into an obligation to assist a dying person, neither to make recommendations for an assisted suicide.”

**In the third document**, Dr, Arthur Caplan from the Bioethics Center of the Pennsylvania University, states that the guiding principles are necessary, but that those into operation are not enough:

“As concerning the protection against the law, they are fairly good, but they do not specify a thing on how to do such a thing in a human way.”

The source of this problem consists, on the one hand, in the fact that the principles tend to be written by those who support the assistance for death, and, on the other hand, they concentrate especially on the role of the doctor instead of concentrating on the reality of the illness, on the suffering and the death of the patient and the suffering of his family.

**The fourth document** explores **the alternatives available to the sick persons that are in terminal phase**. The conclusion was that all the clinic personnel must apply the management standards for pain and interruption of life support. The terminal sedation and the interruption of the water and food supply, although legal, are exceptional options, that are to be taken into consideration only if there are no other acceptable alternatives.

**The fifth document explores the differences between the assisted suicide** and the extraordinary options– terminal sedation and interruption of water and food supply.

**Lois Snyder**, lawyer at the Bioethics Center of the Pennsylvania University states that:

“We think that the difference in what concerns the causes or the arguments that justify the doctor’s intention is not sufficiently significant in order to represent a reason for delimitation.”

“We centered our attention on the argument concerning the corporal integrity. In the assisted suicide we speak about actually doing something, that is the opposite to the avoidance of an undesired invasion of the body.”

We expect new explanations concerning the orientation directions for those who take part in the assisted suicide under doctor guidance, on one or another side, as this practice will be extended, as it would become legal in many countries.

Such a discussion cannot be over, so it is impossible to come to an end. Yet it is required, that, after the presentation of the pro elements versus the cons ones, to suggest a sort of personal opinion.

The right to die is more and more demanded all over the world, in the name of human dignity and by the fear of an unending agony, of a useless suffering.

In order to obtain the legalization of the euthanasia economic arguments were often involved: the limited budget, the high cost of the treatment for the aged, for the patients in terminal phases and for those with genetic or pathologic disabilities.

Those who oppose euthanasia bring forth many arguments: the diminishing of the faith in a doctor, the possibility of a diagnosis error, the loss of human lives, the death of sick people who could be saved later on, the possibility of a thoughtless decision taken by the patient.

The points of view that led to the considering of euthanasia were essentially autonomous, coming from different domains: philosophical, religious, ethical, medical, economic and legal. The cultural level, the religious influences and the growth of the volume of medical knowledge were the factors that modified over time the attitude towards euthanasia.

In the second half of the century, euthanasia became a “fashionable” issue for the medical societies, for the pro-life and pro-choice humanitarian organizations, for the clergy and for the legislative powers in different states.

Rephrasing a famous joke one can say: “Euthanasia is a problem far too serious to be let in the hands of the doctors.”

The decision over the legalization of some forms of euthanasia may be taken only after extended public discussions on the matter. The issue is not only that the members of the society trust or do not trust their doctors. It is obvious that the doctors themselves meet difficulties not only when taking decisions in personal cases, but also when they express their personal opinions on other cases that shorten the human life. The fact that the legal and ethic regulations must be precise and detailed is valuable not only for the patients but first of all, for the doctors themselves.

The public discussions on the opportunity of the legalization of the euthanasia are not only “golden topics” for sociologists and fashionable “press nonsense”, but are vital necessities of societies under reform, in one way or another, maybe on very different criteria.

As the worldwide experiments show, simple solutions, that may be applied swiftly and are easily accepted, do not exist. Not even today and nor in the near future, can we expect a conformity of ideas regarding the practical decisions connected to euthanasia.

It seems that nowadays there is a major gap between what the laws request and what happens in reality, in such a way that we may say that we experience a social hypocrisy at the highest level.

Voluntary euthanasia must not be legalized for the time being. In our society just like in the western ones, in spite of the fact that we live in a new millennium, we are pressed by an open discrimination, a hardly controlled racism and a utilitarian insensibility to vulnerable persons. That is why we cannot have an easy-going attitude of the kind “be it according to your will!” towards the issue of legalization of the euthanasia and the assisted suicide.

A law that forbids its practice must be in use, as it remained in fact in Holland up to 2001, and every aspect must be analyzed according to the specific circumstances. It is not the same thing, if we consider the interruption of reanimation for somebody, by following an order of not doing it, and the case of an interruption done at the whim of the doctor on duty who considers that the patient belongs to a social level or race that is considered by him valueless.

The legal precedent will create a legal source, but the situations will not be abusively generalized, and the people will not be assigned to groupings following a teleological criterion (of estimated result). No law, be it as generous as possible, will ever answer to the infinite variety of the human and clinic situations. Certainly, there will be cases, in which it will be justified or at least tolerated, from the ethical point of view, to set forth the moment of death.

Some fear that, without a clear law, the doctors will never be sure that they will not be accused of murder, if they fulfill the desire of an autonomous patient. Therefore, even if they consider that the voluntary euthanasia is justifiable from the ethical point of view, they will not do it due to the fear of legal consequences.

A tolerant legislation that will favor euthanasia risks opening a way to severe abuses. Few countries have the economic and social conditions, the health care conditions and the traditions of Switzerland and Holland. Therefore, we cannot agree with those who want to generalize this thing, wherever and whenever, without taking into consideration the local circumstances. It is recommendable that those who have access to different administration of recipes for death to be hold under control.

The patients must be offered all the pain alleviance methods and the control of the disabling symptoms available at the moment (e.g. marijuana, and so on and so forth), even appealing to energetic balancing by using acupuncture or other alternative therapies (complementary therapies).

Anyhow, the change in the attitude towards death and agony offers an excellent paradigm with concern to the way in which the ever-changing therapies force us to consider the significant changes in the value system and in the social institutions.

I am convinced that the treatment of this extremely controversial subject will generate many discussions, interpretations and commentaries.

I fully agree with those who consider that the controlled death, established and planned by euthanasia, implies at least two sides.

The first side is assumed by the “applicant”, who wishes to end up the suffering and the pain, by getting ahead of his destiny.

There are many, who, when sane and when their life flows normally, consider this issue with a sheer resolution that is applied in a terminal phase. They want this out of two reasons:

- one is that there is no point to suffer, when death is certain and unavoidable, and the illness brings about horrible pain

- the other is linked to the family and circle of friends, who also suffer uselessly and are tortured by it.

When destiny brings you in such a situation the things get messed up and torments begin.

Personally, I lived many years near a great Romanian medico-legal personality, who, as he was sane, whenever we discussed the matter, his decision was as clear as possible: to end up life. But, as the cruel fate brought him to a terminal phase, close to agony, in short time, he lost hope. The resolute man, confident in himself, whom I often took part in hunting parties with, who knew the best the duties of his profession and about the aspects of death, when he reached the ultimate month of suffering, on his way to death, accepted a paramedic, a kind of a shaman he called “the saviour”, clinging to every thread or hope for life. Moreover, this happened after many local and international medical committees set the diagnosis of malign digestive tumor with metastasis, which he knew so well.

Death came quickly, pitiless, and he has been conscious to the last moment of his life.

The second side implied in a possible euthanasia is, of course the team of professionals (doctors, psychologists, lawyers, etc.) who design the procedures. Have you ever been part of such a team “of death” there would be no way to avoid some questions one has hardly an answer.

Is it right and moral to kill out of compassion? The doctor dressed in white, according to the Hippocratic Oath, is obliged to save the life and not to intentionally kill. What if immediately after a killing out of compassion a miraculous medicine were discovered, turning the untreatable illness into a curable one? What if the person afflicted, in a moment of despair, today wished the euthanasia and tomorrow regretted it? Will God punish this decision taken by both sides implied in the euthanasia? In order to obtain the pardon, which is part of the religious dogmas, we have to follow the redemption through suffering as the Saviour did on the cross.

Personally, I believe in a free and decent life in which we have the possibility of selecting the way to follow in life, without being compelled, by separating the right for wrong. The decision must be our own and have to be respected. I am thinking this way now as I write these lines, maybe because now I am sane. Tomorrow? Nobody knows! There is just one thing I know – today I must live intensely….
